# Involvement of Rev1 in alkylating agent‐induced loss of heterozygosity in *Oryzias latipes*

**DOI:** 10.1111/gtc.12746

**Published:** 2020-02-05

**Authors:** Yoshihiro Fujikawa, Tomoko Ishikawa‐Fujiwara, Tony Kuo, Norio Shinkai, Tatsuma Shoji, Takashi Kawasaki, Yasuhiro Kamei, Yoshiyuki Sakuraba, Ayuko Sato, Masato Kinoshita, Yoichi Gondo, Shunsuke Yuba, Tohru Tsujimura, Jun Sese, Takeshi Todo

**Affiliations:** ^1^ Radioisotope Research Center Institute for Radiation Research Osaka University Suita Japan; ^2^ Radiation Biology and Medical Genetics Department of Genome Biology Graduate School of Medicine Osaka University Suita Japan; ^3^ Japan Society for the Promotion of Science Research Fellow Tokyo Japan; ^4^ RWBC‐OIL, AIST Tokyo Japan; ^5^ Artificial Intelligence Research Center (AIRC), AIST Tokyo Japan; ^6^ Cancer Translational Research Team RIKEN Center for Advanced Intelligence Project Tokyo Japan; ^7^ Division of Molecular Modification and Cancer Biology National Cancer Center Research Institute Tokyo Japan; ^8^ Humanome Lab., Inc. Tokyo Japan; ^9^ Functional Biomolecular Research Group Biomedical Research Institute National Institute of Advanced Industrial Science and Technology (AIST) Ikeda Japan; ^10^ Spectrography and Bioimaging Facility National Institute for Basic Biology Okazaki Japan; ^11^ Genomic Sciences Center (GSC) RIKEN Yokohama Institute Yokohama Japan; ^12^ Department of Pathology Hyogo College of Medicine Nishinomiya Japan; ^13^ Division of Applied Bioscience Graduate School of Agriculture, Kyoto University Kyoto Japan; ^14^ Present address: Department of Molecular Life Sciences Tokai University School of Medicine 143 Shimokasuya Isehara 259‐1193 Japan

**Keywords:** alkylating agent, chemical mutagenesis, DNA damage, DNA polymerase, loss of heterozygosity, mutagenesis, translesion synthesis

## Abstract

Translesion synthesis (TLS) polymerases mediate DNA damage bypass during replication. The TLS polymerase Rev1 has two important functions in the TLS pathway, including dCMP transferase activity and acting as a scaffolding protein for other TLS polymerases at the C‐terminus. Because of the former activity, Rev1 bypasses apurinic/apyrimidinic sites by incorporating dCMP, whereas the latter activity mediates assembly of multipolymerase complexes at the DNA lesions. We generated *rev1* mutants lacking each of these two activities in *Oryzias latipes* (medaka) fish and analyzed cytotoxicity and mutagenicity in response to the alkylating agent diethylnitrosamine (DENA). Mutant lacking the C‐terminus was highly sensitive to DENA cytotoxicity, whereas mutant with reduced dCMP transferase activity was slightly sensitive to DENA cytotoxicity, but exhibited a higher tumorigenic rate than wild‐type fish. There was no significant difference in the frequency of DENA‐induced mutations between mutant with reduced dCMP transferase activity and wild‐type cultured cell. However, loss of heterozygosity (LOH) occurred frequently in cells with reduced dCMP transferase activity. LOH is a common genetic event in many cancer types and plays an important role on carcinogenesis. To our knowledge, this is the first report to identify the involvement of the catalytic activity of Rev1 in suppression of LOH.

## Graphical abstract

LOH is a common genetic event in many cancer types and plays an important role on carcinogenesis. We found defect in the catalytic activity of rev1 exhibited a higher tumorigenic rate and high frequency of LOH. To our knowledge, this is the first report to identify the involvement of the catalytic activity of Rev1 in suppression of LOH.

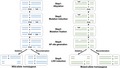

## 1 INTRODUCTION

Genomic DNA undergoes constant modifications mediated by endogenous and environmental genotoxic agents. If these modifications are not repaired, they block DNA replication by the high‐fidelity DNA polymerases Polδ and Polε. The resultant stalled replication forks have deleterious consequences on genome stability, inducing insertions and deletions, chromosome number alterations and chromosome aberration (Gaillard, Garcia‐Muse, & Aguilera, 2015; Techer, Koundrioukoff, Nicolas, & Debatisse, 2017; Zeman & Cimprich, 2014). This genome instability leads to loss of heterozygosity (LOH) and ultimately triggers carcinogenesis. To circumvent this catastrophic scenario, cells possess specialized translesion synthesis (TLS) DNA polymerases that replace stalled replicative DNA polymerases and resolve replication blocks by inserting a nucleotide opposite the modified base on the template (Sale, Lehmann, & Woodgate, 2012). However, TLS DNA polymerases frequently incorporate incorrect nucleotides, leading to point mutations. Thus, TLS polymerases protect the genome from the dangerous consequences of blocked replication at the expense of introducing point mutations.

Several TLS enzymes have been identified in vertebrates, including the Y‐family DNA polymerases Rev1, Polη, Polι, and Polκ, and the B‐family DNA polymerase Polζ. TLS polymerase‐mediated bypass of DNA damage is carried out via a two‐step process (Livneh, Ziv, & Shachar, 2010; Prakash & Prakash, 2002). In the first step, an “inserter” polymerase incorporates a nucleotide across the lesion. In most cases, this step is catalyzed by Y‐family DNA polymerases, and each TLS polymerase is specialized for bypass of a specific type of DNA lesion. In the second step, an “extender” polymerase extends the aberrant DNA primer terminus. This step is catalyzed by the B‐family DNA polymerase Polζ.

Although Rev1 belongs to the Y‐family of DNA polymerases, it is unique in its deoxycytidyl transferase activity (Haracska, Prakash, & Prakash, 2002; Nelson, Lawrence, & Hinkle, 1996). Rev1 can use only dCTP as a dNTP source, and thus can incorporate dCMP opposite to G‐templates, and can also bypass apurinic/apyrimidinic (AP) sites or G‐adducts. dCMP transferase activity is achieved by unique mechanisms for template recognition and incorporation of incoming dCTP. In addition to the polymerase core domain, Rev1 possesses a loose α‐loop structure, termed the N‐digit, at the N‐terminal side of the core domain. The incoming dCTP forms hydrogen bonds with an arginine residue in the N‐digit (Nair, Johnson, Prakash, Prakash, & Aggarwal, 2005). The SRLH motif in the N‐digit, which contains the arginine residue, is well conserved in Rev1 across various species, and replacement of amino acid residues in this motif markedly reduces dCMP transferase activity (Piao, Masuda, & Kamiya, 2010).

Despite several reports of dCMP transferase activity in in vitro systems (Nelson, Gibbs, Nowicka, Hinkle, & Lawrence, 2000; Otsuka, Kunitomi, Iwai, Loakes, & Negishi, 2005), the involvement of dCMP transferase activity in AP site bypass in vivo is controversial. Alkylating agent reacts with DNA bases to generate a variety of covalent adducts, which are cytotoxic and also mutagenic. Guanine is the target base and is most frequently alkylated by alkylating agents. Because base alkylation generally weakens the N‐glycosyl bond, alkylation of guanine leads to spontaneous depurination and appearance of AP sites (Verna, Whysner, & Williams, 1996). Bypass of the G‐derived AP site with Rev1 dCMP transferase activity does not result in mutation. On the other hand, Polζ, Polδ and Polη also can bypass the AP site and generate a C>A transversion by incorporating A opposite the AP site (Haracska et al., 2001). Thus, the involvement of Rev1 in AP site bypass is obscured by error‐free bypass by Rev1 and mutagenic bypasses by other polymerases. However, many studies have demonstrated the relevance of the dCMP transferase activity in in vivo systems (Auerbach, Bennett, Bailey, Krokan, & Demple, 2005; Kim, Mudrak, & Jinks‐Robertson, 2011; Nelson et al., 2000).

In addition to the N‐digit domain, Rev1 is unique among Y‐family polymerases in that it possesses an extra C‐terminal domain (CT domain) through which other TLS polymerases interact with Rev1. Rev1 acts as a scaffold in recruitment of other TLS enzymes to DNA lesions and mediates assembly of multipolymerase complexes (Guo et al., 2003; Nelson et al., 2000; Ohashi et al., 2004; Tissier et al., 2004).

Translesion synthesis polymerase deficiency can lead to UV‐induced mutations and skin cancer (Masutani, Kusumoto, Iwai, & Hanaoka, 2000). However, the potential contribution of TLS dysfunction to other human cancers is unknown. On the other hand, recent studies have identified involvement of TLS pathways in carcinogenesis. TLS polymerases generate many of the mutational signatures present in cancer cells (Rogozin et al., 2018), and the TLS pathway is pathologically activated in many cancers (Gao et al., 2018, 2016). These findings indicate that TLS may have diverse roles in tumor suppression. To identify the mechanisms underlying the tumor suppressive effects of the TLS pathway, a simple vertebrate model system that can complement mammalian systems would be of significant utility. Small laboratory fish such as zebrafish and medaka are highly useful for both molecular and classical genetics. In particular, medaka has a compact genome and thus is suitable for genomic analyses (Wittbrodt, Shima, & Schartl, 2002). Thus, in the present study, we used medaka to study TLS‐mediated mutagenesis and response to alkylating DNA damage. Alkylating agent diethylnitrosamine (DENA) is an extremely potent liver carcinogen in rodent (Verna et al., 1996), and its carcinogenicity has also been reported in medaka (Brown‐Peterson, Krol, Zhu, & Hawkins, 1999; Ishikawa, Shimamine, & Takayama, 1975; Lauren, Teh, & Hinton, 1990; Liu, Kullman, Bencic, Torten, & Hinton, 2003; Nakazawa, Hamaguchi, & Kyono‐Hamaguchi, 1985; Teh & Hinton, 1998). We generated three types of mutations in the *rev1* gene, a null‐allele, a CT domain‐deleted allele and a catalytic activity‐reduced allele. We then evaluated the sensitivity of each mutant to the alkylating agent DENA. The catalytic activity‐reduced mutant exhibited mild sensitivity to DENA toxicity, whereas the null and CT domain‐deleted alleles were hypersensitive to DENA toxicity. Genomic analysis by next‐generation sequencer (NGS) has enabled not only the detection of mutations, but also analysis of their chromosomal distribution. Loss of heterozygosity (LOH) is a common genetic event in cancer and is known to be involved in the somatic loss of tumor suppressor gene (Vogelstein et al., 2013; Weinberg, 1991). NGS analysis of the chromosomal distribution of induced mutations revealed that LOH was common in the catalytic activity‐reduced mutant.

## 2 RESULTS

### 2.1 Disruption of *rev1* in medaka fish

**Figure 1:**
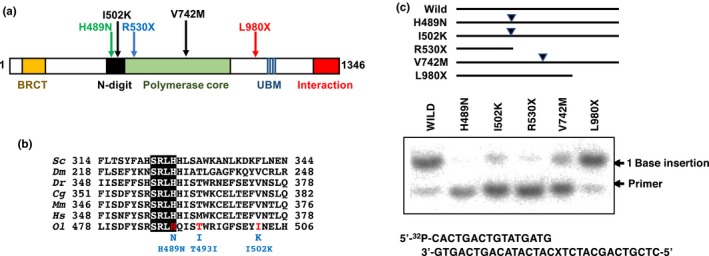
Mutations identified in the *rev1* gene. (a) Protein structure of Rev1 and mutations changing the coding region of the *rev1* gene. Amino acid changes are indicated by arrows above the structure. (b) Alignment of the amino acid sequence of the N‐digit domain from several organisms. Corresponding sequences for *Saccharomyces cerevisiae* (Sc), *Drosophila melanogaster* (Dm), *Danio rerio* (Dr), *Galus galus* (Gg), *Mus musculus* (Mm), *Homo sapiens* (Hs) and *Oryzias latipes* (Ol) Rev1 are shown. The SRLH motif is highlighted with a black box, and the identified amino acid substitutions in medaka mutants are indicated by red characters. (c) dCMP transferase activity of mutant Rev1. Nucleotides were inserted opposite of template AP sites by purified wild‐type and mutant Rev1. The upper panel represents the schematic illustration of wild‐type and mutant Rev1. The lower panel represents the sequences of the primers and the damage‐containing template. X indicates the position of AP site analogue. A weak one base insertion band was detected in the R530X lane, which might be contamination of *Escherichia coli*‐derived polymerase activity in the cell extract

By screening the medaka Target Induced Local Lesion in Genome (TILLING) library (Taniguchi et al., 2006), we identified two non‐sense mutations of *rev1*, each of which resulted in the generation of a premature stop codon at either residue arginine 530 (R530X) or leucine 980 (L980X). R530X lost both the catalytic activity and the C‐terminal interaction domain of *rev1*, whereas the catalytic domain remained in L980X (Figure 1a and Figure S1). In addition to the two non‐sense mutants, we identified three or one mis‐sense mutations in the N‐digit region (H489N, T493I and I502K) or in the polymerase core domain (V742M), respectively. The SRLH motif in the N‐digit region is well conserved in Rev1 in various species (Figure 1b), and several studies report that this motif is essential for the catalytic activity of Rev1 (Nair et al., 2005; Piao et al., 2010). The H489N mutation is positioned at this motif (Figure 1b). To determine whether this motif was also essential in medaka, we determined the catalytic activity of recombinant H489N protein with an in vitro primer extension assay using oligonucleotides containing the AP site analogue as a template. H489N and R530X lost dCMP transferase activity, whereas the activity remained in L980X (Figure 1c). In this study, we used R530X as the null mutant, L980X as the CT domain deletion mutant and H489N as the catalytic activity‐reduced mutant.

### 2.2 In vitro sensitivity to UV and DENA

**Figure 2:**
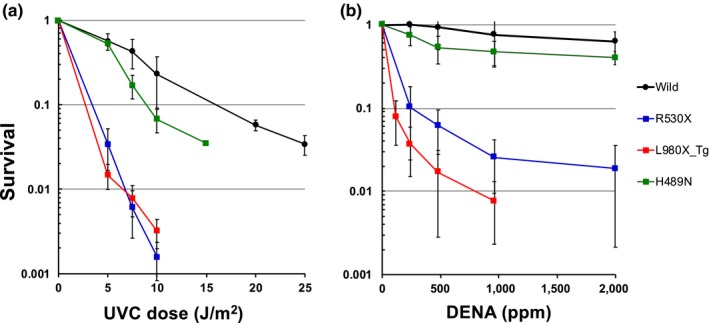
Survival curve of wild‐type and mutant cells after UV irradiation or DENA exposure. Survival of cultured cells in response to (a) UV radiation or (b) DENA exposure was determined by a colony formation assay. For the L980X mutant, to avoid the effect of RNA degradation by non‐sense‐mediated mRNA decay (Baker & Parker, 2004), cultured cells derived from transgenic medaka, in which the BAC carrying the L980X mutation in the *rev1* gene was introduced into the R530X mutant, were used. The mean and standard deviation (*SD*) of three independent experiments are shown. For UV irradiation, wild versus L980X‐Tg or R530X were significant (*p* < .01), and wild versus H489N is significant at the dose of 7 J/m^2^ (*p* < .01), but not significant at 10 J/m^2^. For DENA treatment, wild versus L980X‐Tg or R530X were significant (*p* < .01), but wild versus H489N were not significant. Dunnett's test was used

To determine the sensitivity of these mutants to genotoxic agents at the cellular level, we established cultured cell lines from mutant embryos. UV radiation and the alkylating agent DENA were used as genotoxic agents. UV is a commonly used genotoxic agent, and *Rev1*‐deficient mutants are highly sensitive to UV in several model organisms. DENA is a representative chemical carcinogen with the potential to cause tumors in various organs, including the liver. R530X and L980X mutants were highly sensitive to 254 nm UV irradiation, with a fivefold increase in sensitivity relative to the wild type, whereas H489N mutants exhibited milder sensitivity and were approximately 1.5‐fold more sensitive than the wild type (Figure 2a). The cytotoxic effects of DENA on null or CT domain deletion mutants were remarkably strong. Whereas the H489N mutant was twofold to threefold more sensitive to DENA exposure than the wild type, the R530X and L980X mutants were over 10‐fold more sensitive than the wild type (Figure 2b and Figure S2).

### 2.3 In vivo sensitivity to DENA

**Figure 3:**
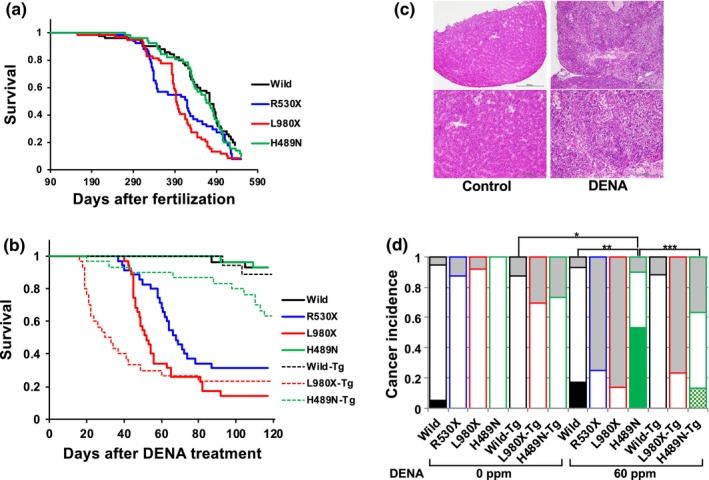
DENA sensitivity of medaka fish. (a) Life spans of wild‐type and mutant fish, as represented by a Kaplan–Meier survival curve. Wild type (*n* = 50), R530X (*n* = 51), L980X (*n* = 59) and H489N (*n* = 51). Time is shown as days after fertilization. Wild versus R530X or L980X were significant (*p* < .01), but wild versus H489N is not significant. Generalized Wilcoxon test was used. (b) Kaplan–Meier survival curves of wild‐type (*n* = 29), R530X (*n* = 35), L980X (*n* = 29), H489N (*n* = 29), Wild‐Tg (*n* = 18), H489N‐Tg (*n* = 30) and L980X‐Tg (*n* = 30) fish after 60 ppm DENA exposure for 2 weeks. Time is shown as days post‐DENA treatment. Wild versus R530X, L980X, L980X‐Tg or H489N‐Tg, and R530X versus H489N‐Tg were significant (*p* < .001), but wild versus H489N or Wild‐Tg were not significant. Generalized Wilcoxon test was used. (c) Representative images of livers stained with hematoxylin and eosin. Left images are from control untreated fish, and right images are from DENA‐treated fish (H489N, 60 ppm for two weeks). Magnified images of the boundary region between normal tissue and tumor tissue in livers from DENA‐treated fish (lower right) and the similar region in livers from control fish (lower left) are shown. (d) Incidence of liver tumors in wild and mutant fish 4 months post‐DENA treatment. The bar for each genotype consists of three categories: “tumor‐bearing” (filled with black, green or halftone green), “tumor free” (blank) and “unknown” (gray). As individual fish died before the fourth month of post‐DENA treatment have not been checked for liver tumor, these individuals were categorized as “unknown.” Untreated control fish: wild (*n* = 19), R530X (*n* = 24), L980X (*n* = 25), H489N (*n* = 29), wild‐Tg (*n* = 16), L980X‐Tg (*n* = 23) and H489N‐Tg (*n* = 30). DENA‐treated fish: wild (*n* = 29), R530X (*n* = 36), L980X (*n* = 36), H489N (*n* = 30), wild‐Tg (*n* = 17), L980X‐Tg (*n* = 30) and H489N‐Tg (*n* = 30). For H489N fish untreated control (0 ppm) versus DENA‐treated (60 ppm; *p < .001), and for DENA‐treated fish wild versus H489N (**p = .0021) and H489N versus H489N‐Tg (***p = .01) were significant, but for wild fish 0 ppm versus 60 ppm and for DENA‐treated fish wild versus wild‐Tg or H489N‐Tg were not significant. These statistical processes were carried out excluding “unknown.” Chi‐squared test was used. The numbers of male and female fish used or survived at the end of observation are summarized in Table S2

Next, we examined DENA toxicity in adult fish. The life span of H489N was the same as the wild type, whereas R530X and L980X mutants exhibited slightly shorter life spans. However, all genotypes survived over 10 months (Figure 3a). Thus, 4‐month‐old fish were used for DENA toxicity studies. Fish were exposed to 60 ppm DENA for 2 weeks, and survival was monitored for the subsequent 4 months. Consistent with the sensitivity of cultured cells, both R530X and L980X mutants were hypersensitive to DENA (Figure 3b). At 35 days post‐treatment, the survival of both mutants decreased rapidly, and only 10%–30% of fish survived after 4 months post‐treatment. On the other hand, more than 90% of wild‐type and H489N fish survived after 4 months post‐treatment. At 4 months post‐treatment, all surviving fish were killed and examined histologically for the onset of liver tumors (Figure 3c). In R530X and L980X mutants, liver tumor was not detected in the surviving individuals. In the H489N mutant, liver tumor could be detected in 60% of surviving individuals, and the tumor incidence rate was higher than that of wild‐type fish, 20% of surviving individuals (Figure 3d). To confirm that the DENA sensitivity of each mutant was caused by *rev1* mutation, transgenic (Tg) lines were established in which BAC clones carrying either wild‐type, H489N or L980X mutations of *rev1* were introduced into the R530X mutant. The wild‐type Tg line fully complemented the phenotype of R530X in the cytotoxicity (Figure 3b). H489N‐Tg partially complemented the cytotoxicity (Figure 3b), potentially because of the relatively lower expression of *rev1* in the H489N‐Tg line than wild‐type Tg line (Figure S3). As fish died before fourth month post‐treatment have not been checked for liver cancer, they are categorized as “unknown” and indicated as gray bar in Figure 2d. The early‐dead individuals are expected to have no developed tumor, since the first six weeks after the start of DENA treatment are cytotoxicity stage in the wild strain (Lauren et al., 1990). However, we cannot exclude the possibility that strong cytotoxicity of DENA has enhanced the tumor development in R530X and L980X mutant.

### 2.4 Genomic analysis of alkylating agent‐treated *rev1* mutant cells

**Figure 4:**
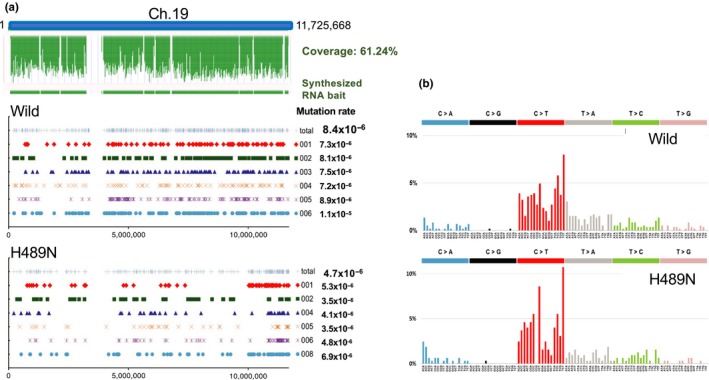
Frequency, spectrum and chromosomal localization of DENA‐induced mutations. (a) Distribution of the identified mutations, lined up along the left arm of chromosome 19. Cells of each genotype were treated with DENA at a dose yielding 2%–5% survival (for the wild type 6,000 ppm for 12 hr and for H489N 3,000 ppm for 12 hr), and genomic DNA was extracted from single colonies. The left arm of chromosome 19 was enriched with synthesized RNA bait and subjected to NGS. Sequencing was conducted for six colonies. The top panel indicates chromosomal location of the RNA bait (green). The lower two panels indicate the chromosomal locations of the identified mutations in wild‐type and H489N cells. In each panel, the lower six lines indicate the positions of identified mutations. The top line indicates the sum of identified mutations. Mutation frequencies are indicated to the right of each line. (b) Mutational signatures of DENA‐induced mutations. Frequencies of substitution mutations in each genotype are shown. The profiles are displayed using the 96‐substitution classification, which is defined by reporting the specific base substitution combined with the immediate neighboring 5′ and 3′ nucleotides

Next, we examined the mutagenic effects of DENA on wild‐type and H489N cells using NGS. Chromosome 19 is the smallest chromosome in medaka and thus is suitable for high‐throughput deep sequencing. We carried out genomic analysis of the left arm of chromosome 19 by target‐enrichment sequencing and determined spectrum and chromosomal distribution of induced mutations (Figure 4). Cells of each genotype were exposed to DENA at a dose that resulted in 2%–5% survival. After culturing for 4 additional days post‐treatment, cells were collected and thinly reseeded to form separate colonies, and genomic DNA was extracted from each separate colony (Figure S4). Genomic analysis was carried out for six clones of each genotype. Although there were variations in the chromosomal locations of mutation induction among individual clones, when the results of the six clones were aggregated, there was no significant difference in mutation location between the two genotypes (Figure 4a). The mutation frequency was higher in wild type (8.4 × 10^–6^) than H489N (4.7 × 10^–6^). The mutational profile of the induced mutations is summarized as the mutational signature for each genotype in Figure 4b. The mutational signature was similar among the two genotypes, with the C>T transition being most common.

**Figure 5:**
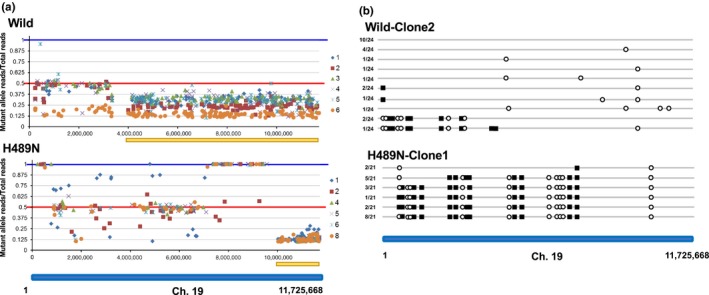
Chromosomal distribution of mutations and LOH. (a) Proportion of mutant alleles lined up along the left arm of chromosome 19. For each mutation identified by NGS, the value obtained by dividing the number of mutant allele reads by that of the total reads was mapped on the left arm of chromosome 19. Values obtained from each individual colony are distinguished by a different color. The vertical axis represents the value of mutant allele reads/total reads, and the horizontal axis represents the number of bases from the top of chromosome 19. Pale yellow bars indicate the regions including the location where the peak calling value of mutant alleles was lower than that of normal alleles. (b) Distribution of LOH on the left arm of chromosome 19. A colony with a most drastic “mutant allele proportion” fluctuation pattern was selected from 6 clones in each genotype (wild type: Clone2 and H489N: Clone1), and 21 or 24 independent subcolonies were recovered by reseeding cryopreserved cells. Mutations present in each subclone were identified for 33 or 34 chromosomal positions by capillary sequencing (Figure S5). Several positions in each subclone were homozygous for mutant or normal allele, and the positions were defined as LOH. Each subclone could be divided into several LOH patterns, and these LOH patterns are shown. The patterns included homozygous for mutant allele (black square) and normal alleles (white circle). The rate of clones having the distribution pattern of the corresponding LOH is indicated at the left of each bar

Because induced mutations are introduced into one of the two chromosome copies, the identified mutations are expected to be heterozygous between the normal allele and the mutant allele. However, we found that the proportions of mutant allele were not necessarily 50% and exhibited various values (Figure 5a). Of particular interest was when only mutant alleles were detected, which is indicative of LOH in the corresponding chromosomal region. Interestingly, the appearance of mutant homo‐alleles was most common in the H489N mutant.

The bias of the proportion of mutant alleles could have been due to heterogeneity in the genomic composition of individual cells in each population. To clarify this, single cell‐derived clones were prepared from the original cell population. We generated single cell‐derived colonies again from the original cell population by reseeding cryopreserved cells (Figure S4) and detected mutant alleles in each colony by capillary sequencing. The results are summarized in Figure S5. Not all clones were heterozygous for mutant and normal alleles, but some clones contained positions where mutant or normal alleles were homozygous, and their distribution on the chromosome varied between clones. As expected, the induced mutations were heterogeneous in the original population. The location of normal or mutant homozygous allele appears to be where LOH occurs. Based on the results of capillary sequencing (Figure S5), the chromosomal pattern of LOH distribution was summarized and schematically illustrated in Figure 5b. In the wild type, 14 of 24 clones exhibited LOH, whereas in H489N all 21 clones exhibited LOH.

**Figure 6:**
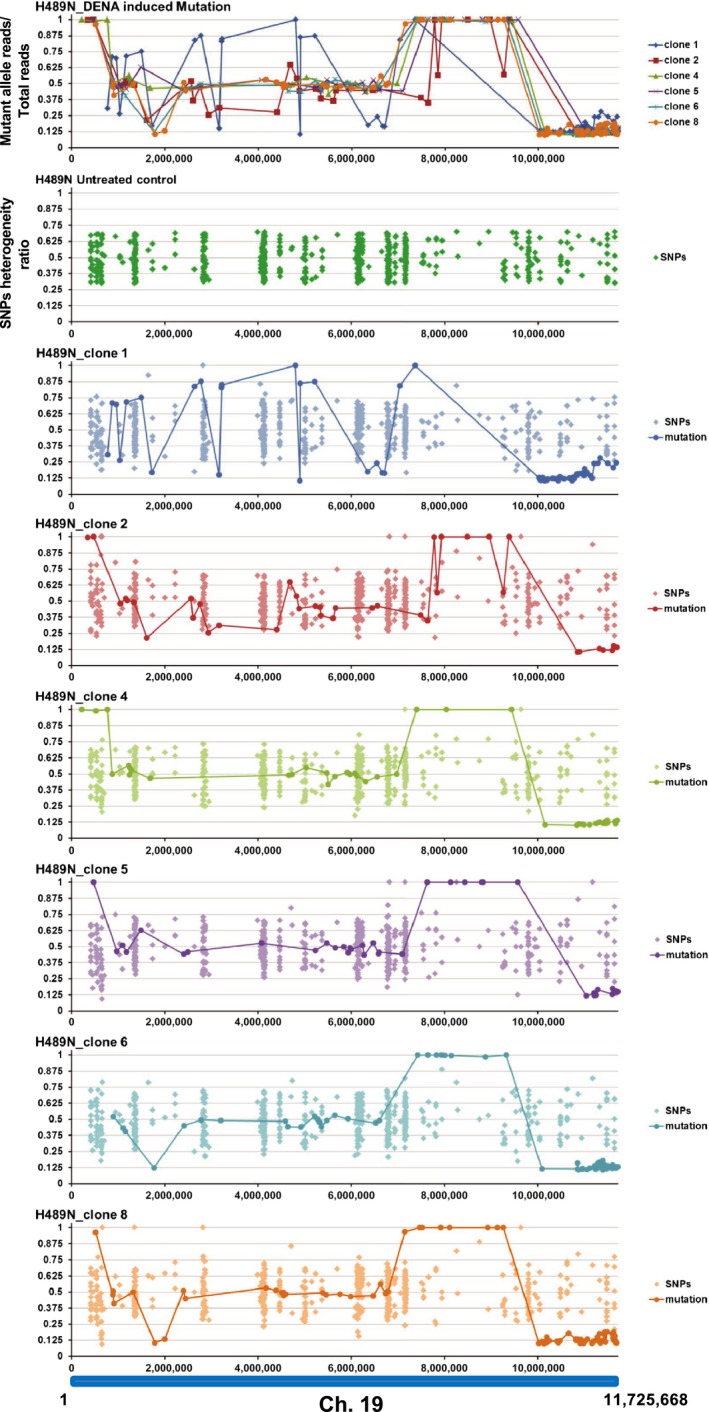
Variation of SNPs in each H489N clone. Fluctuations of the heterogeneity ratio of SNPs in each clone are shown. SNPs were defined as the positions in which the base calling by NGS was heterogeneous in untreated control cells. For each SNP, the heterogeneity ratio was calculated in each clone and mapped on chromosome 19. Top panel: chromosomal distribution of the proportion of mutant alleles in each H489N clone (as shown in Figure 5a). Each dot represents the value of number of mutant allele reads/number of total reads. They are mapped on chromosome 19 and connected by a line. Second panel: chromosomal distribution of SNPs detected in untreated control cells. The vertical axis indicates the heterogeneity ratio of each SNP. Lower six panels: chromosomal distribution of heterogeneity ratio of each SNP in each clone. The darker color symbols connected by a line indicate the identified mutations, and the lighter color symbols indicate the SNPs. The vertical axis indicates both the value of number of mutant allele reads/number of total reads for induced mutations and the heterogeneity ratio of SNP

To clarify whether neighboring LOHs occurred independent to each other, next we examined whether LOH were linked to nearby SNP fluctuation. The mutants used in this study were obtained by screening of TILLING medaka library. As TILLING library was generated by treatment of inbred Cab strain with chemical mutagen (Ishikawa et al., 2010; Taniguchi et al., 2006), the mutant obtained has a vast amount of SNPs in addition to the mutation at gene of the interest. Although the identified mutants were back‐crossed to Cab strain more than 5 generations to remove unexpected mutations, many SNPs still remain in the genome of mutants as well as cultured cell established from each mutant. The positions in which the base calling by NGS was heterogeneous in untreated control cells were used as SNPs. The criteria of heterogeneity are the position in which one allele appears in 33%–67% of the other allele. The heterogeneity ratio of each SNP was calculated for each of the 6 clones in the DENA‐treated cells and mapped on chromosome 19 (Figure 6). In DENA‐treated cells, the heterogeneity ratio was more discrete than that of untreated cells, and some hetero‐allele positions changed to homo‐alleles. However, the LOH in induced mutations did not correlate with the LOH in SNPs.

On the other hand, for some mutagenized bases, underlined with yellow bars in Figure 5a, the percentage of the base calling as mutant alleles by NGS was lower than that of normal alleles. Capillary sequencing of these regions revealed that all bases were heterogeneous between the mutant and normal alleles, but that the peak of normal base calling was higher than that of mutant bases (denoted by pale yellow shading in the table shown in Figure S5). Although the height of the peak of capillary sequencing does not necessarily accurately indicate the number of template bases, the ratio of the height of peak was also roughly correlated with that of the read depth by NGS. Biased peak heights were commonly observed between the two genotypes, but the cause for this unexpected observation is not clear at present.

## 3 DISCUSSION

Alkylating agents are a major class of chemotherapeutic drugs. The primary mechanism of action is cytotoxic DNA damage, but these agents also cause mutagenic damage (Fu, Calvo, & Samson, 2012; Verna et al., 1996). In the present study, we demonstrated that *rev1* null (R530X) and CT domain deletion (L980X) mutants were hypersensitive to the alkylating agent DENA in cytotoxicity. Contrastingly, the catalytic activity‐reduced mutant (H489N) exhibited only a slight increase in DENA cytotoxicity, but exhibiting increased tumorigenicity relative to the wild type.

DENA is an SN1‐type alkylating agent that reacts with nitrogen ring structures and extracyclic oxygen molecules of DNA bases to generate a variety of covalent adducts (Fu et al., 2012; Verna et al., 1996). The most predominant ethylating adducts on nitrogen rings are N7‐ethyl guanine (7etG), followed by N3‐ethyladenine (3etA). 7etG, by itself, is neither cytotoxic nor mutagenic, but is prone to AP site formation by spontaneous depurination. 3etA is highly cytotoxic, as this adduct inhibits DNA polymerases. AP sites and 3etA can be bypassed by a combination of several TLS polymerases. Polη and Polζ are involved in bypass of 3meA (Johnson, Yu, Prakash, & Prakash, 2007; Monti et al., 2014), and Polδ and Polη can bypass AP sites in cooperation with the extender function of Polζ (Haracska et al., 2001; Zhao, Xie, Shen, & Wang, 2004). Rev1 interacts with these polymerases at the CT domain and acts as a scaffold protein at the stalled replication fork. Thus, the DENA hypersensitivity of the L980X mutant could be due to functional defects of Rev1 as a scaffold protein. Another alternative possibility is the subcellular localization of the L980X‐Rev1 protein. L980X‐Rev1 also lacks nuclear localizing signal (NLS; Figure S1). We cannot exclude the possibility that the inability to translocate to the nucleus is responsible for the DENA hypersensitivity of the L980X mutant.

**Figure 7:**
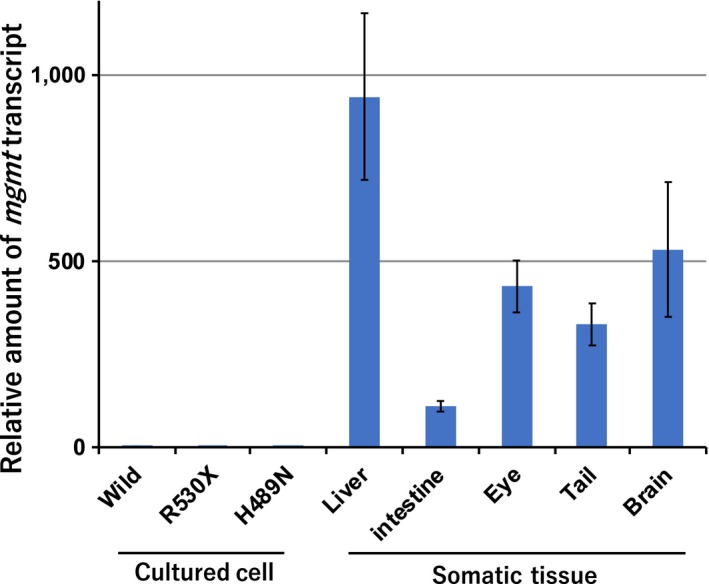
*mgmt* expression in cultured cells and somatic tissues. Reverse transcription and quantitative PCR were conducted using RNA isolated from cultured cells (wild, R530X or H489N) or wild‐type tissues (liver, intestine, eye, tail or brain). The mean and standard deviation (*SD*) of three assays using three different samples are shown

In contrast to cytotoxicity, the spectrum of induced mutations was not significantly different between *rev1* mutant and the wild type. In both genotypes, the C>T transition occurred most frequently. O^6^‐ethyl guanine (O^6^etG) is the main adduct of extracyclic oxygen mediated by DENA. O^6^etG is more mutagenic than the nitrogen adducts, as O^6^etG can readily mispair with thymine during DNA replication to generate C>T transition mutations (Fu et al., 2012). The relative abundance of O^6^meG produced by other SN‐1 type alkylating agents, including *N‐*methyl‐*N′*‐nitrosourea (MNU) and *N*‐methyl‐*N′*‐nitrosoguanidine (MNNG), is 10% of that of the most abundant alkylated adduct, 7meG. MMS, an SN‐2 type alkylating agent, produces O^6^meG adducts at a frequency less than 1% that of 7mtA. Compared with these alkylating agents, DENA produces many more O^6^etG adducts at approximately 50% of the abundance of 7etG (Verna et al., 1996). The relative abundance of O^6^etG adducts could contribute to the high frequency of C>T transitions induced by DENA. Another possible cause of abundant C>T transitions is differences in the repair activity of O^6^etG. Alkylated guanine at the O^6^ position is repaired by the O^6^‐methylguanine‐DNA methyltransferase (MGMT) repair enzyme (Fu et al., 2012). *Mgmt* is conserved in most species, but its activity varies between species and tissues. *Mgmt* expression is modulated primarily by epigenetic control. Hypermethylation of CpG islands on the *Mgmt* gene promoter significantly silences the *Mgmt* gene (Dunn et al., 2009). We found that *mgmt* expression was extremely low in the cultured medaka cells used in the present study compared with other medaka tissues (Figure 7). The high frequency of C>T transition is presumed to be due both to the abundant production of O^6^etG and to the low repair activity in cultured medaka cells.

**Figure 8:**
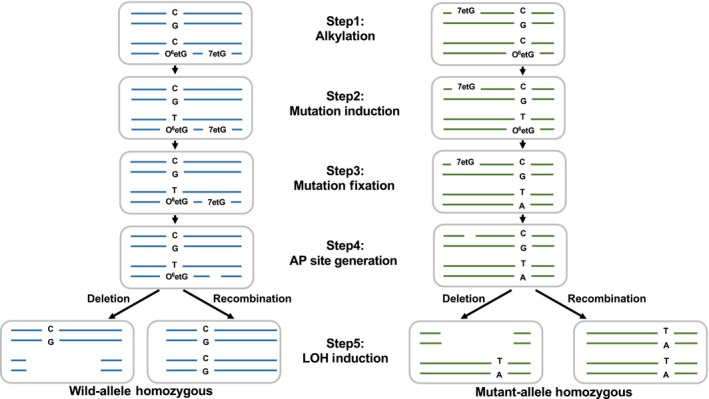
LOH induction model. Proposed LOH induction model is shown. Step1: DENA generate alkylated base in genome DNA, Step2: mutations are induced at the opposite of O^6^etG, Step3: induced mutations are fixed subsequent replication, Step4: AP site are generated at the remaining 7etG by spontaneous depurination during subsequent several rounds of cell division and Step5: LOH are induced at some AP site

While deficiency in the catalytic activity of *rev1* (H489N mutant) only modestly affected the cytotoxicity and mutagenicity of DENA, LOH was induced at high frequency in H489N cells exposed to DENA. The average number of LOH per cell for the wild type or H489N was 2.2 or 17.8, respectively. Although each colony used for NGS analysis was derived from a single cell, the chromosomal LOH distribution was heterogeneous in each colony, suggesting that LOH induction persisted for several cell cycles after seeding DENA‐treated cells. The persistence of LOH induction for several cell cycles after seeding suggests that some DNA lesions still remained in the genomic DNA after cell division and that LOH occurred in the remaining DNA lesions. AP sites resulting from these lesions might be responsible for LOH induction in the H489N mutant. 7etG is a good candidate for the DNA lesion responsible for induction of LOH, as 7etG is not cytotoxic and thus remains after cell division, which eventually generate AP site by spontaneous depurination. The LOH induction model described is shown in Figure 8. Interesting hypothesis is that Rev1 could participate in the pathway choice between the TLS pathway and LOH induction, and the tendency toward LOH might increase when the bypass pathway is inhibited, such as in the H489N mutant. On the other hand, we cannot exclude another possibility, in which DNA damage induced genomic instability persisted for a while, thereby inducing LOH.

However, in the H489N mutant, defects other than dCMP transferase activity must also be taken into account. Mutants lacking dCMP transferase activity in yeast or DT40 cells are not sensitive to UV (Ross, Simpson, & Sale, 2005), whereas H489N mutant cells exhibited mild UV sensitivity in the present study, suggesting that the H489N mutant had unknown defects in Rev1 activities. In fact, 5′‐deoxyribose phosphate lyase activity was recently identified in the catalytic core domain of Rev1 (Prasad, Poltoratsky, Hou, & Wilson, 2016). Rev1 may have unknown activities, which are defective in the H489N mutants. Further functional analyses of the H489N mutants are an ongoing topic of investigation.

Loss of heterozygosity is a general term that includes both LOH with copy number losses and copy number neutral. The former is caused by deletion and the latter by somatic recombination. Three potential pathways are proposed for mitotic recombination in somatic cells, including reciprocal crossover, gene conversion and break‐induced replication. In the present study, DENA‐induced LOH did not correlate with nearby SNP fluctuation (Figure 6). This suggests that each LOH occurred independently, and that long tract gene conversion, break‐induced replication or large deletion was not involved in LOH. Single cell genomic analyses will determine the type of recombination occurring and also clarify whether deletion is involved in DENA‐induced LOH. These are important topics of future investigation.

To evaluate the effects of Rev1 on DENA genotoxicity, we used cultured cells. However, the lower expression of *mgmt* in cultured cells than in somatic tissues indicates that mutagenesis in cultured cells is not a good model for carcinogenesis, especially in the liver, which has high *mgmt* expression. Thus, future studies of hepatic mutagenesis are necessary to clarify the contribution of genotoxicity to liver carcinogenesis. LOH plays an important role in carcinogenesis. The present study newly identified increased LOH in *rev1* mutants and provides an excellent in vivo system to study the mechanisms of LOH induction and also the role of LOH in carcinogenesis.

## 4 EXPERIMENRAL PROCEDURES

### 4.1 Medaka maintenance

The inbred Cab medaka line was used. Fish were maintained as described previously (Ishikawa et al., 2010). All experiments were carried out in accordance with the Japanese laws and guidelines for the care of experimental animals and according to the Osaka University Animal Experimental Rules. All animal protocols were approved by the Committee on the Ethics of Animal Experiments of Osaka University (Approval Number: 27‐047‐001). All surgical procedures were carried out under tricaine methanesulfonate (MS‐222) anesthesia, and all efforts were made to minimize suffering.

### 4.2 Generation of medaka mutants

To generate mutant fish, the medaka TILLING library (Ishikawa et al., 2010; Taniguchi et al., 2006) was screened using temperature gradient capillary electrophoresis (TGCE) as described previously (Sakuraba et al., 2005). Fifteen base substitution mutants were obtained (Figure S1). The obtained mutant fish of interest were out‐crossed with wild‐type fish of the inbred Cab strain for more than five generations. Genotyping was carried out by a combination of the HRM assay and capillary sequencing as described previously (Ishikawa et al., 2010). The sequences of all oligonucleotides used in this study are listed in Table S1.

### 4.3 In vitro primer extension assay using recombinant protein

A Medaka *rev1* cDNA clone (Ole17.04a) was obtained from NBRP Medaka. Amino acid substitutions at H489N, I502K or V742M or non‐sense mutations at R530X or L980X were introduced into *rev1* cDNA by a two‐step PCR using primers encoding the mutated nucleotide. The entire coding sequences of wild or mutant cDNA were cloned in‐frame with the glutathione S‐transferase gene in the pGEX4T vector (Amersham Biotech.). Over‐expression and preparation of crude cell extracts were conducted as described previously (Kobayashi et al., 2000). GST‐fusion proteins were purified using a glutathione‐Sepharose 4B column (Amersham Pharmacia Biotech), and fractions with GST activity were pooled and applied to a PD‐10 gel filtration column (Amersham Pharmacia Biotech) to remove the glutathione present in the buffer (50 mM Tris, pH 8.0). Fractions containing recombinant protein, as determined by SDS‐PAGE, were pooled and used for the primer extension assay.

The primer extension assay was conducted as described previously (Ishikawa et al., 2001). A 16‐mer DNA primer (5′‐CACTGACTGTATGATG) and a complementary 30‐mer primer (5′‐CTCGTCAGCATCTXCATCATACAGTCAGTG), where the underlined X is placed with the abasic analogue (Masutani et al., 2000), were used. The labeled template primer was used at a final concentration of 4 nM. Reaction mixtures contained 50 mM Tris–HCl (pH 8.0), 2 mM dithiothreitol, 50 mM KCl, 5 mM MgCl_2_, 100 ng/ml bovine serum albumin, 0.1 mM dCTP and 250 ng of partially purified enzyme in a final volume of 5 µl. The reaction was incubated at 37°C for 1 hr and terminated by addition of an equal amount of 2× loading buffer. Reactions were separated using a 14% polyacrylamide gel containing 8 M urea and visualized by exposing the gel to X‐ray film.

### 4.4 Establishment of cultured cell and clonogenic assays

Primary cultured cell lines were derived from embryos of each mutant as described previously (Ishikawa et al., 2010; Kobayashi et al., 2000). Cells were cultured in L‐15 medium supplemented with 10% fetal bovine serum (Gibco BRL), 50 mg/ml streptomycin, 50 U/ml penicillin and 10 mM HEPES (pH 7.5) at 27°C. Uniformly growing cell lines were obtained after repeated passages. Clonal cells were used for experiments.

Cells were assayed for colony‐forming ability using the feeder‐layer method as described previously (Ishikawa et al., 2010). For UV irradiation, cells were irradiated with UVC with a GL‐10 germicidal lamp (Toshiba) at the fluence rate of 0.6 J m^2^ s^1^ as determined by a UVX radiometer (Ultra‐Violet Products Inc.). After UV irradiation, cells were cultured in 6‐well plates in the presence of feeder cells (7 × 10^4^ cells/well) pre‐irradiated with 100 Gy γ‐ray. Cells were cultured for 10 days, and colony‐forming units were counted after staining with crystal violet. For DENA treatment, DENA was activated with an S9 liver homogenate mixture (S9MIXTS, IEDA Trading Co.) prior to cell exposure. S9 mix was added to L15 culture medium containing DENA kept at 37°C, such that the final concentration of S9 was 5%. One milliliter of culture medium containing activated DENA was added to cells in each well of the 6‐well plates. After treatment with DENA for 6 or 12 hr at 27°C, cells were washed with PBS and displaced by L‐15 culture medium containing feeder cells (7 × 10^4^ cells/well) and cultured for 10 days. Colony‐forming units were counted after staining with crystal violet. The plating efficiency of the wild‐type, R530X, L980X or H489N cells was 0.3%–1.1%, 0.6%–1.8%, 0.1%–1.1% and 0.1%–1.1%, respectively.

### 4.5 Generation of transgenic medaka

For complementation of mutant, the BAC clone (Md0156O24; Matsuda et al., 2001), which covers 11,542,792–11,774,473 of chromosome 21 and contains the whole *rev1* gene (chromosome 21:11,548,478–11,563,126), was used. To identify germ line‐transmitted fish, the *Gfp* gene under control of the *Ol β‐actin* promoter (*β‐act‐Gfp*) was introduced into the BAC clone using two unique *AscI* (11,602,182) and *FseI* (11,771,804) restriction enzyme recognition sites. Because a smaller BAC clone is convenient for further manipulation and establishment of transgenic fish, we replaced the large genomic sequences between 11,602,182 and 11,771,804 with the *β‐act‐Gfp* fragment. The DNA fragment containing *β‐act‐Gfp* between the two restriction sites was amplified from *pβ‐act‐Gfp* (Bubenshchikova et al., 2005) and inserted into Md0156O24. The resultant BAC was named BAC‐Rev1‐wild‐type. BAC clones carrying the H489N or L980X mutation were generated from BAC‐Rev1‐wild‐type by recombineering (Zhang, Muyrers, Testa, & Stewart, 2000) and named as BAC‐Rev1‐H489N or BAC‐Rev1‐L980X (Figure S7). To prevent non‐sense‐mediated mRNA decay (Baker & Parker, 2004), the BAC‐Rev1‐L980X was designed to have only one stop codon by deleting the region from 980th amino acid to the stop codon. Recombineering was carried out using a Counter‐Selection BAC Modification Kit (Gene Bridge GmbH) according to manufacturer's instructions. Each BAC DNA was isolated and purified using a Qiagen Large‐Construct Kit and was used to generate BAC transgenic fish. Each BAC clone was injected into R530X mutant fish fertilized egg, and a transgenic line was established. After injection of each BAC clone, three lines were established for BAC‐Rev1‐wild‐type (Wild‐TgO8F, Wild‐TgO14F and Wild‐TgO42F) and for BAC‐Rev1‐L980X (L980X‐TgG13F, L980X‐TgG13M and L980X‐TgG7F), whereas for BAC‐Rev1‐H489N, one Tg line (H489N‐Tg) was established. *rev1* expression in each Tg line was determined by RT‐quantitative PCR analysis of the liver from each Tg line (Figure S3). Wild‐TgO14F, L980X‐TgG13F and H489N‐Tg were used for experiments.

### 4.6 DENA treatment of fish

The life span of R530X and L980X mutant fish was slightly shorter than that of wild‐type or H489N mutant fish (Figure 3a). However, almost all fish survived and were healthy more than 10 months after hatching. Thus, for fish studies, experiments were designed such that all procedures were completed within 8 months after hatching. A group of 4‐month‐old medaka was exposed to DENA (Sigma‐Aldrich) for 2 weeks at a concentration of 60 ppm, with the water changed once weekly. After exposure, the fish were transferred to clean water and held in a tank for an additional 24 hr. Both unexposed control fish and exposed fish were subsequently transferred to separate, flow‐through aquaria maintained on a recirculating system. Four months after DENA treatment, fish were anesthetized with tricaine methanesulfate, and livers were excised and fixed in Bouin's solution. Fixed tissues were embedded in paraffin blocks. Cross‐sections were cut at a thickness of 5 µm and stained with hematoxylin–eosin. Sections were examined and photographed using light microscopy. The presence of liver tumors was determined by observing the section with the maximum cutting surface of the liver.

### 4.7 Target capture and next‐generation sequencing

After DENA treatment and subsequent culture for 4 days, cells were collected and replated on new dishes at a sufficient dilution to allow single cells to form separate colonies (Figure S4, upper half). As a control, solvent‐treated cells were also replated for single colony isolation. Six and two single colonies were isolated from DENA‐treated and control cells, respectively, and genomic DNA was extracted from each clone. Genomic DNA on the left arm of chromosome 19 from each sample was enriched with RNA baits using a Sure Select Custom Kit (Agilent Technologies) according to the manufacturer's instructions. The RNA baits were designed to cover 61.24% of the left arm of chromosome 19 (nucleotides 1–11,725,668). Genomic DNA sequencing libraries were prepared using the Agilent Sure Select QXT Reagent Kit (Agilent Technologies). The genomic DNA sequencing library consisted of eight samples (six DENA‐treated and two untreated), in which each sample was distinguished by index and used for NGS (HiSeq2000 (R530X), HiSeq2500 (Wild and H489N) and HiSeq4000 (WGS) Illumina Inc.). The detection of induced mutations by target enrichment was almost the same as that of whole‐genome sequencing (Figure S6).

### 4.8 Sequencing data analysis

After removal of the terminal adaptor sequences and low‐quality data, reads were mapped to the reference medaka genome with BWA MEM (0.7.12‐r1039). Based on the mapped reads, small insertions and deletions and single‐nucleotide variants in the captured DNA were identified in control and DENA‐treated samples using GATK (v3.3‐0) software. To distinguish mutations found in control samples from those in the DENA‐treated samples, the log‐likelihood ratio (LLR) of each SNP was calculated using EAGLE software (Kuo, Frith, Sese, & Horton, 2018), representing the confidence level of the mutation being caused by DENA exposure. Variants with high LLR indicated a high possibility that the mutation was caused by DENA exposure. These candidates were validated by capillary sequencing to distinguish genuine mutations from sequencing errors and were selected according to the following criteria. Candidate variants were listed by descending LLR value, and capillary sequencing was carried out in batches of ten candidates, from the highest LLR candidates to those with lower scores. Capillary sequencing revealed that all high‐LLR candidates were genuine mutations and revealed the borderline LLR value at which both real and false mutants were mixed. All candidates positioned at the borderline LLR value were validated by capillary sequencing.

### 4.9 Mutational signature analyses

For mutational spectra analysis, induced mutations were annotated by the 96 possible trinucleotide context substitutions as described previously (Alexandrov et al., 2013). The distribution of 5′ and 3′ nucleotides flanking the mutations was calculated directly from the reference genome.

### 4.10 Quantitative PCR

Reverse transcription and quantitative PCR were conducted as described previously (Ishikawa et al., 2010). RNA was isolated from cultured cells or tissue using the Sepasol RNAI Super kit (Nacalai Tesque) and reverse transcribed using the ReverTraAceR qPCR kit (Toyobo). For *mgmt* quantitative PCR, cDNA‐specific primers were designed to amplify either the 5′ or 3′ region of the *Mgmt* transcripts (5′ regions were within exons 2–3, and 3′ regions were within exons 4–5), and *β‐actin* was used for normalization. For *rev1* quantitative PCR, *atm* was used for normalization. As the expression level of *rev1* is relatively low, *atm* with a low expression level was used as control.

### 4.11 Statistical analyses

Statistical analyses were carried out using a BellCurve for Excel. *p*‐Values < .01 were considered statistically significant.

### 4.12 Genome database

MEDAKA1 was used in this study.

## CONFLICT OF INTEREST

The authors declare no competing financial interests.

## AUTHOR CONTRIBUTIONS

Y.F., T.I.‐F. and T.To. designed and managed the study. T.I.‐F., Y.K., T.To., Y.S. and Y.G. performed screening of medaka mutants. Y.F. and T.I.‐F. established and analyzed cultured cells derived from mutant fish. M.K., Y.K. and T.I.‐F. established Tg line. T.K., N.S., T.S., J.S. and T.I.‐F. performed NGS data analysis. A.S., T.I.‐F and T.Tsu. performed histology. T.I.‐F., T.K. and S.Y. performed capillary sequencing. T.I.‐F. performed LOH analysis. Y.F., T.I.‐F. and T.To. wrote the manuscript.

## Data Availability

Genomic DNA sequencing data have been deposited in the DDBJ Sequence Read Archive under accession code, DRA009293 (H489N), DRA009294 (wild), DRA009295 (R530X: WGS) and DRA009296 (R530X).
