# Supplementary material for: Involvement of Rev1 in alkylating agent‐induced loss of heterozygosity in Oryzias latipes
Source: Genes Cells. 2020 Feb 5;25(2):124–38. doi: 10.1111/gtc.12746 (PMC7079036; doi:10.1111/gtc.12746)
Supplement: Supplementary file 1 [file GTC-25-124-s001.pdf]

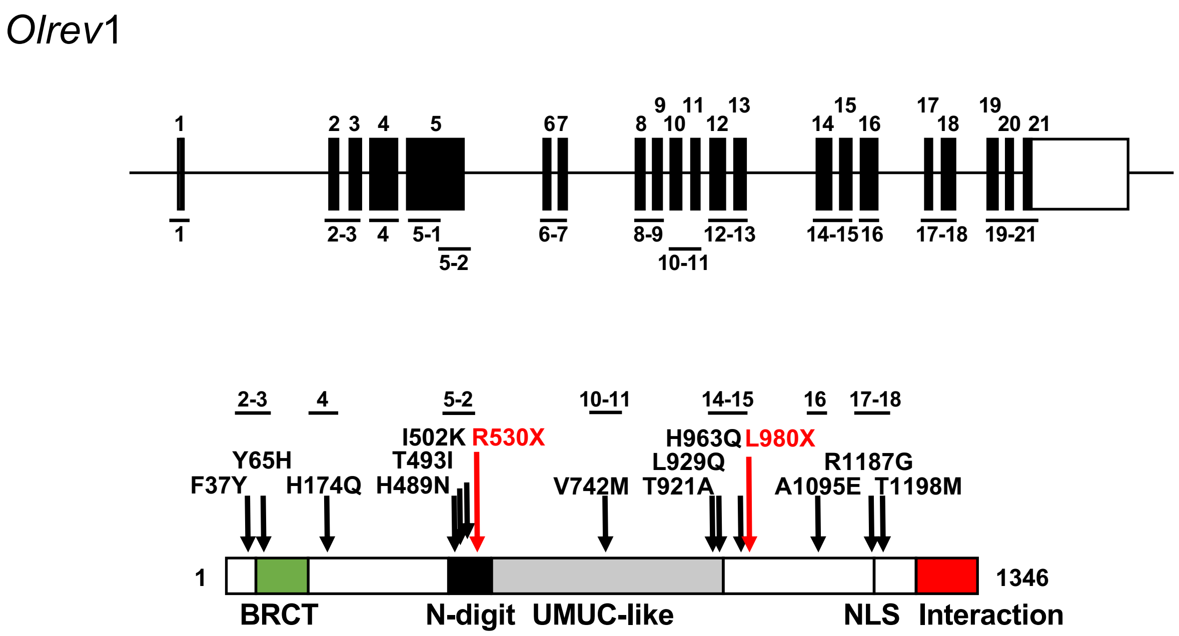

Figure S1. Screening of Rev1 mutations in the TILLING library by TGCE.

Figure S1: Screening of Rev1 mutations in the TILLING library by TGCE. The upper panel represents the intron-exon structure of *rev1*, and the target region is indicated below the panel. Each exon was amplified by PCR using primer sets (Table S1) and analyzed by Temperature Gradient Capillary Electrohoresis (TGCE). The lower panel indicates all mutations detected.

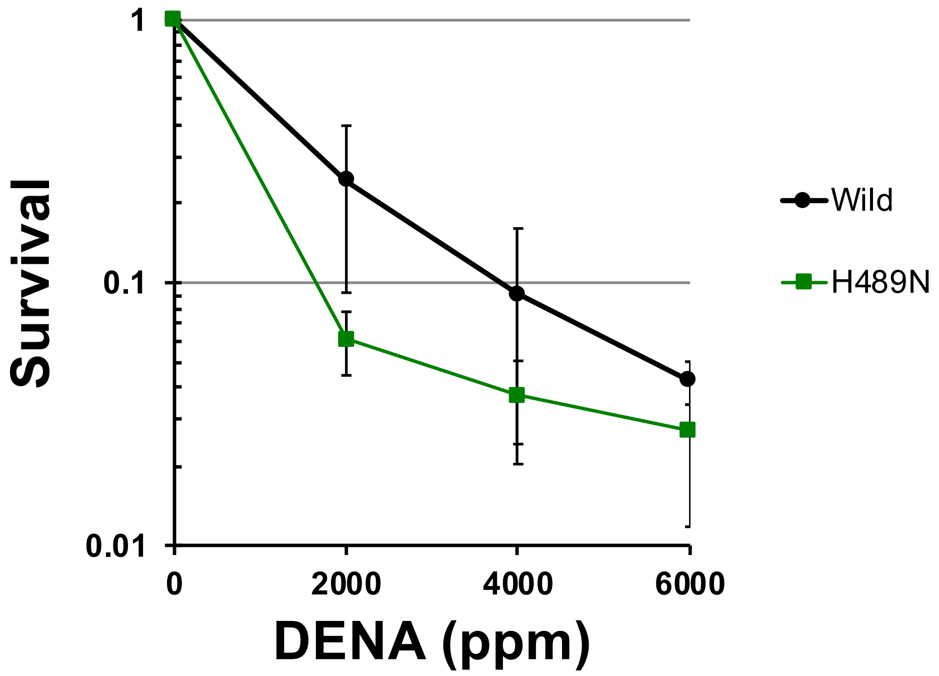

Figure S2. Survival curve of DENA-exposed cultured cells.

Figure S2: Survival curve of DENA-exposed cultured cells.
Cells were exposed to DENA at the specified doses for 12 h.
Wild vs. H489N were not significant.

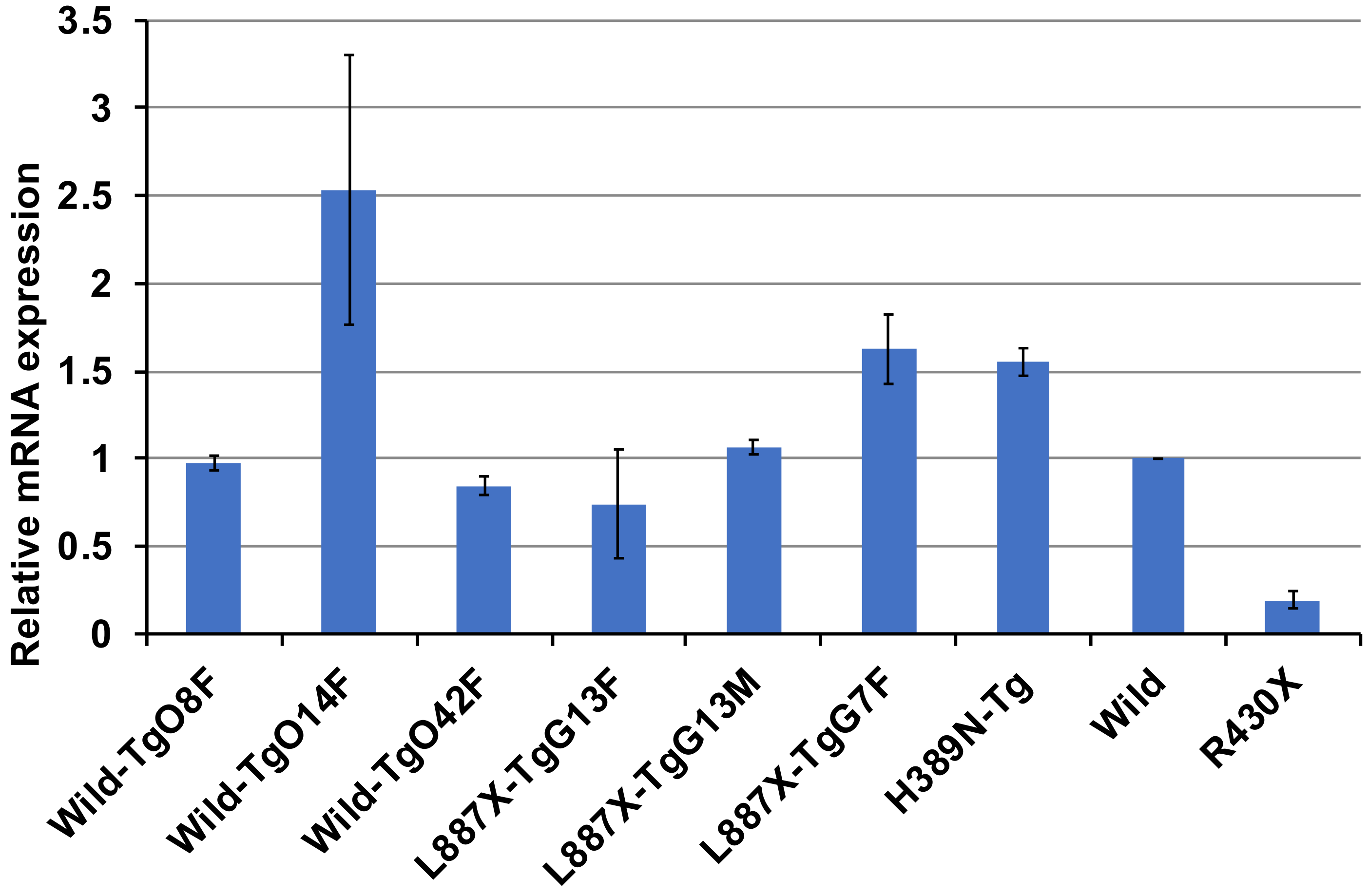

Figure S3. *rev1* expression in Tg lines.

Figure S3: *rev1* expression in Tg lines. Reverse-transcription and quantitative PCR were conducted using RNA isolated from the liver of each Tg line, wild-type fish, or R530X mutant fish. The mean and standard deviation (SD) of the three assays using three different samples are shown.

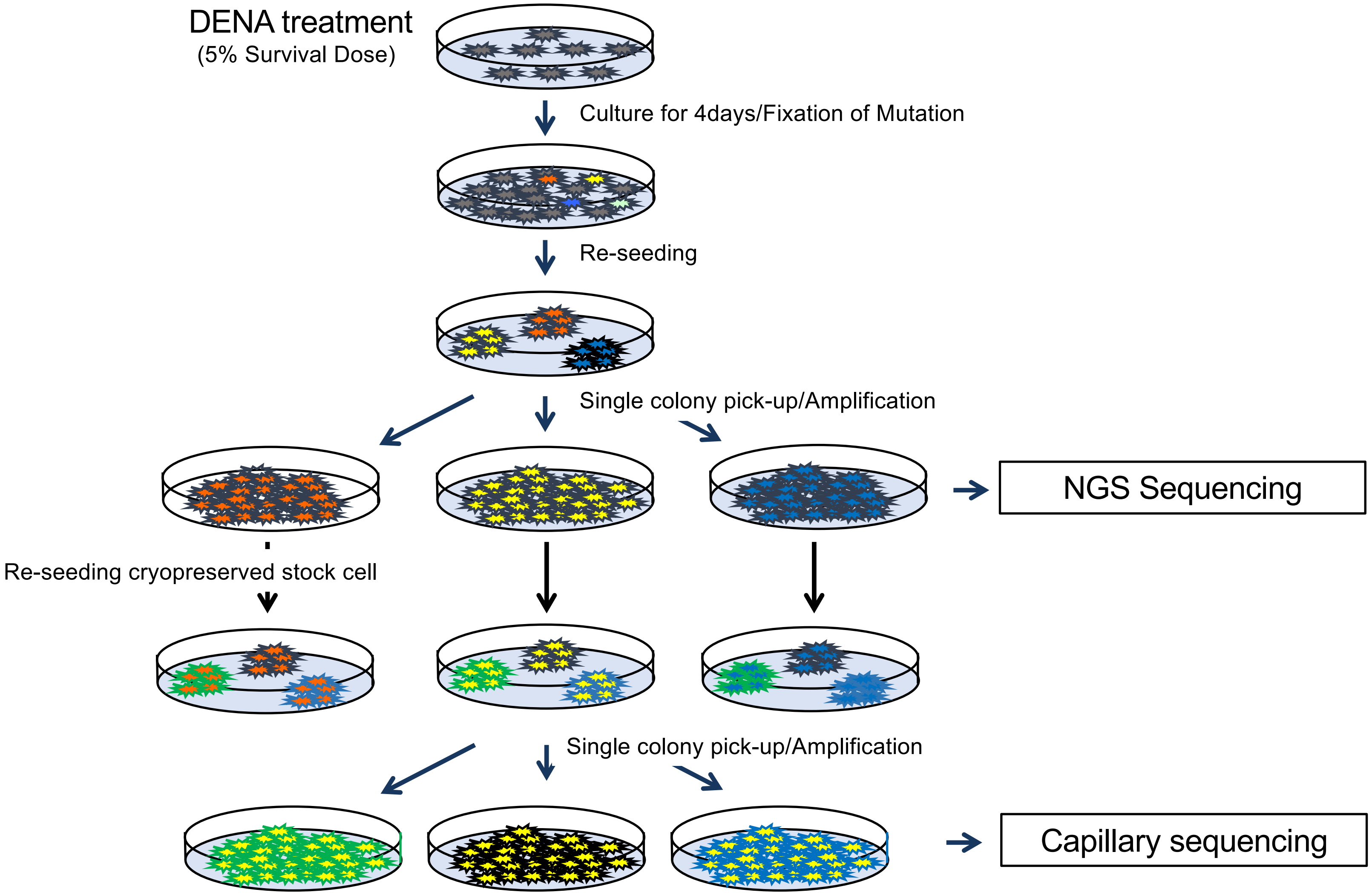

Figure S4. Preparation of single cell-derived colony after DENA treatment.

Figure S4: Preparation of single cell-derived colony after DENA treatment. Cells were exposed to DENA and cultured for 4 days post-treatment. Cells were collected and thinly re-seeded to form separate colonies, and genomic DNA was extracted from a part of each colony, and used for NGS sequencing. The remaining cells were cryopreserved. The cryopreserved cells were re-seeded again to generate separate colonies. Genomic DNA was extracted from each colony, and used for capillary sequencing. We did not add feeder cells at the step of separate colony isolation.

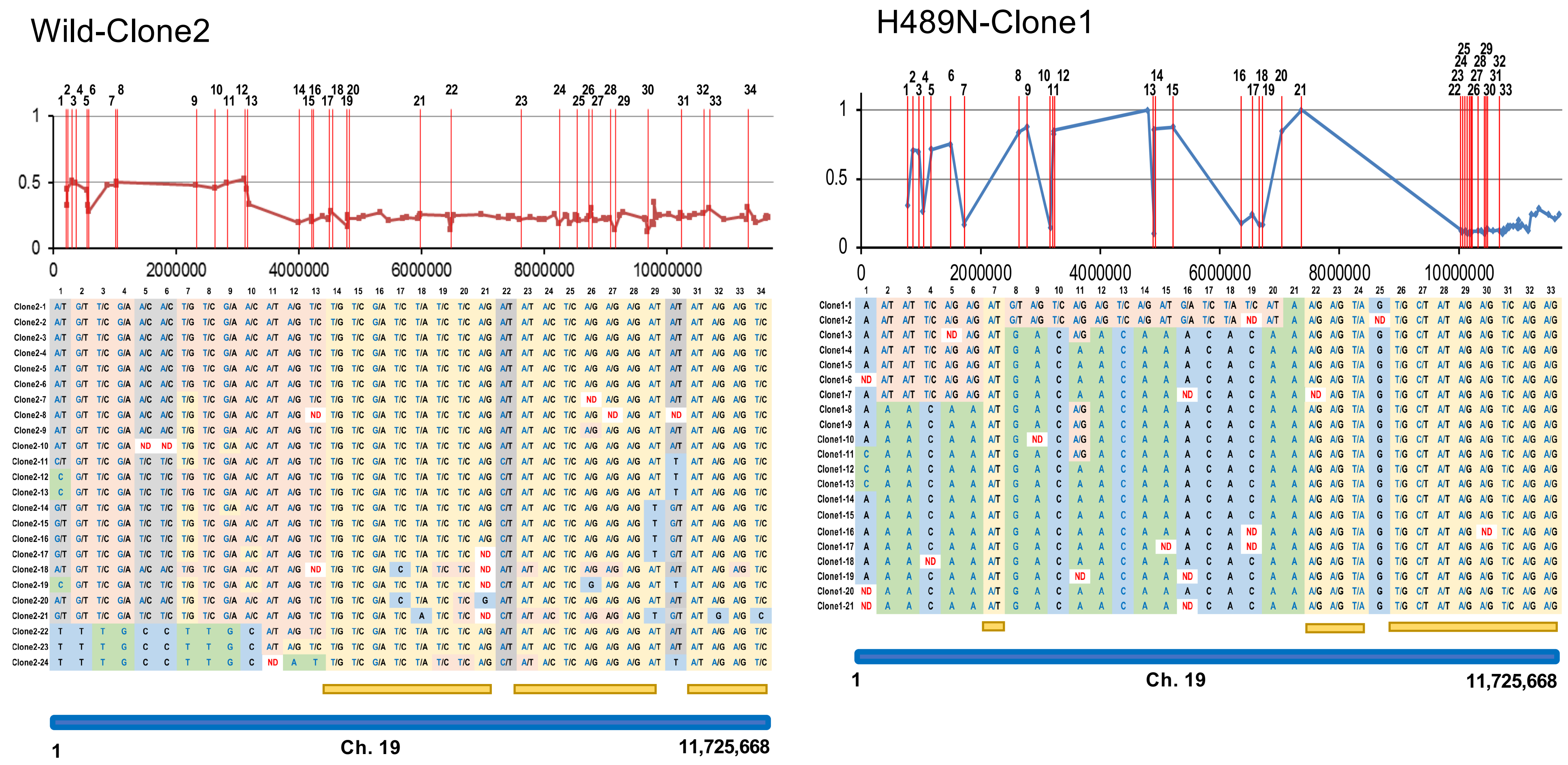

Figure S5. Capillary sequencing of cloned cells.

Figure S5: Capillary sequencing of cloned cells. Based on the results of Figure 5a, one colony showing a most drastic fluctuation pattern was selected for each genotype (Wild-type Clone 2 and H489N Clone 1), and colonies derived from a single cell were generated again by re-seeding cryopreserved cells. 21 or 24 independent colonies were recovered from Wild-type Clone 2 or H489N Clone 1, respectively, and mutations present in each colony were identified by capillary sequencing for 33 or 34 chromosomal positions of Wild-type Clone 2 or H489N Clone 1, respectively. Two sets of panels are shown, including Wild-type Clone 2 (left panel) and H489N Clone 1 (right panel). For each panel, the upper figure shows the chromosomal distribution of the mutant alleles proportion in the selected clone shown in Figure 5a, and the lower table shows the results of capillary sequencing. In the table, mutation-normal heterogeneous position was indicated as mutant base/normal base, and mutation or normal homogeneous position is denoted by a single letter. The positions where the mutant or normal allele was homogeneous are indicated by green or blue, respectively. The positions where the mutant and normal allele were heterogeneous are distinguished by three colors. Pink: the peak height of the base calling of the mutant and the normal allele was the same; light yellow: the base calling of the normal allele was higher than that of the mutant allele; gray: multiple mutations. The numbers at the top of the upper figure and the lower table indicate the position of the mutation. The number assigned to each clone is shown in the left of each table. ND indicates positions in which the sequence were not determined by capillary sequencing. For construction of Figure 5B, each ND was estimated as follows. Wild-type Clone 2: Position 5 and 6 of Clone 2-10 as A/C; Position 11 of Clone 2-24 as A/T; Position 13 of Clone 2-8and -18 as T/C; Position 21 of Clone 2-17, -18, -19, and 2-21 as A/G; Position 26 of Clone 2-7 as A/G; Position 27 of Clone 2-8 as A/G; and Position 30 of Clone 2-8 as A/T. H489N Clone 1: Position 1 of Clone 1-6, -20, and -21 as A; Position 4 of Clone 1-18 as C; Position 5 of Clone 1-3 as A/G; Position 9 of Clone 1-10 as A; Position 11 of Clone 1-19 as A; Position 15 of Clone 1-17 as A; Position 16 of Clone 1-7, -19 and -21 asA; Position 19 of Clone 1-2, -16, and -17 as C; Position 22 of Clone 1-7 as A/G; Position 25 of Clone 1-2 as G; and Position 30 of Clone 1-16 as A/G.

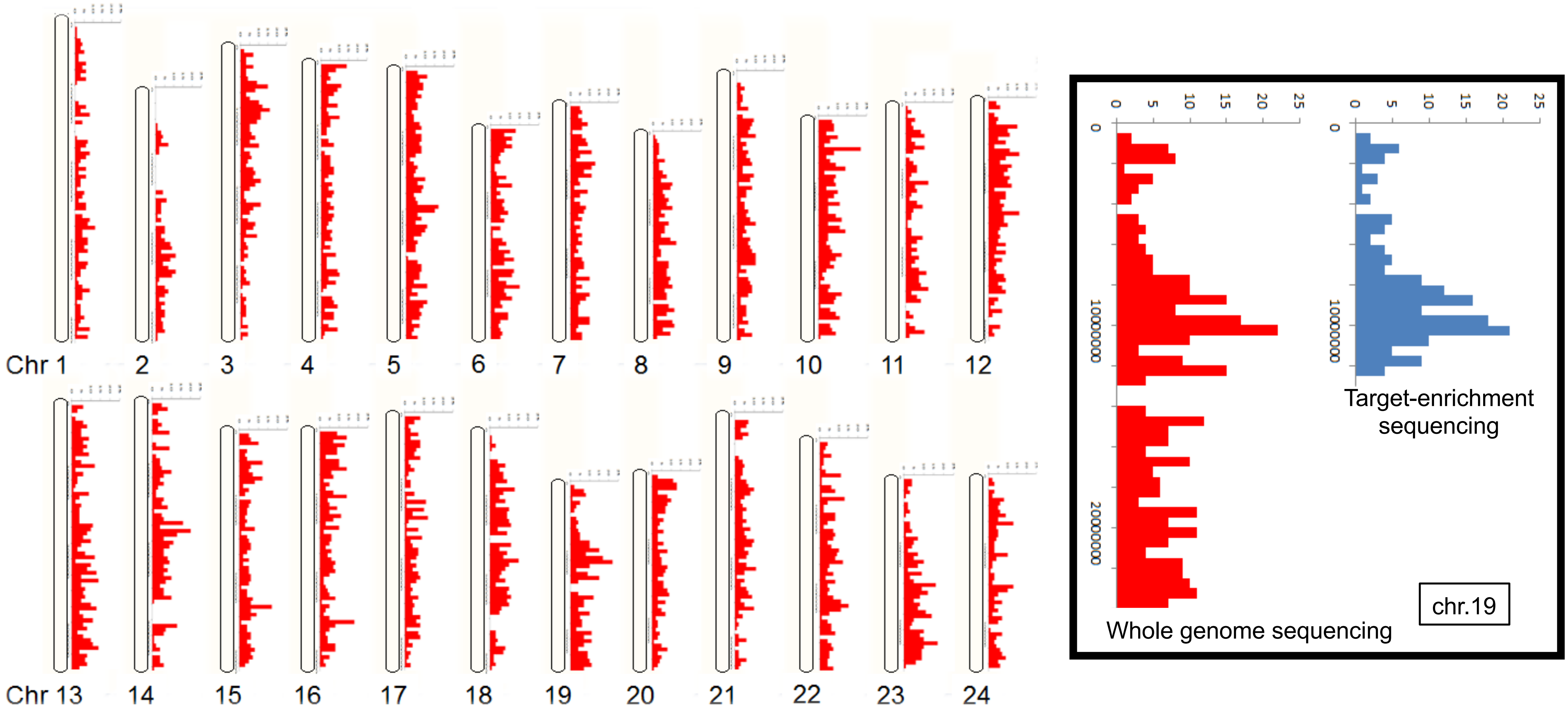

Figure S6. Comparison of identified mutations between target-enrichment sequencing and whole-genome sequencing by NGS.

Figure S6: Comparison of identified mutations between target-enrichment sequencing and whole-genome sequencing by NGS. In the left panel, results of WGS are lined up on each chromosome, and in the right panel, mutations identified by target-enrichment sequencing or whole-genome sequencing are lined up on the left arm of chromosome 19. Results of R530X are shown.

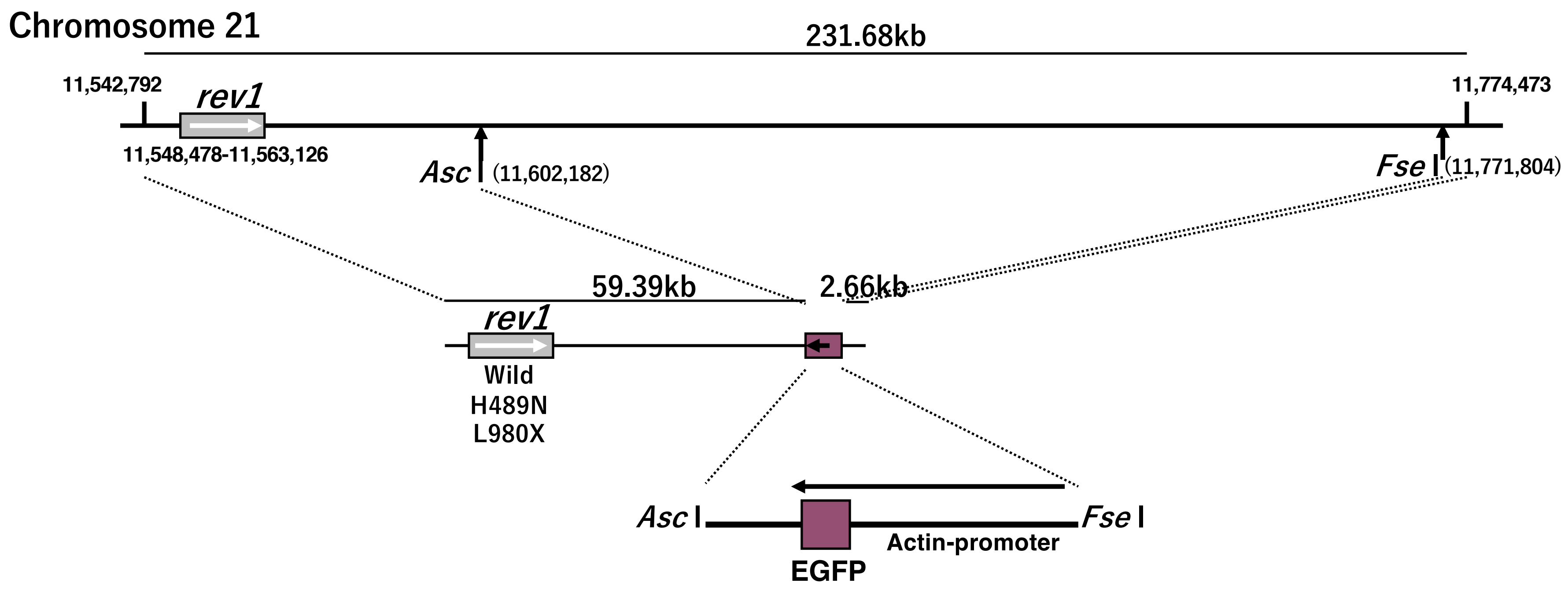

Figure S7. Construction of the BAC-*rev1* vector.

Figure S7: Construction of the BAC-*rev1* vector.
