# Supplementary material for: Involvement of Rev1 in alkylating agent‐induced loss of heterozygosity in Oryzias latipes
Source: Genes Cells. 2020 Feb 5;25(2):124–38. doi: 10.1111/gtc.12746 (PMC7079036; doi:10.1111/gtc.12746)
Supplement: Supplementary file 2 [file GTC-25-124-s002.pdf]

Table S1. Oligonucleotides used in this study.

Primer Name	Sequence
Screening of TILLING library	
OlRev1-exon1-F	CCAGAGCAACAGCACTTTGG
OlRev1-exon1-R	GGGCCCAGTAACAGAAATGC
OlRev1-exon2-3-F	AAACAAGCCCCTTGATGCTC
OlRev1-exon2-3-R	CGATCTGGGCTAAACGGGTA
OlRev1-exon4-F	CATCAGGCCAGAGTGGATCA
OlRev1-exon4-R	GATTTGCAGAAACACCTTGGA
OlRev1-exon5-1-F	AAGATCCAAGGTGTTTCTGCAA
OlRev1-exon5-1-R	TGAGATTGCTTTGGGGGACT
OlRev1-exon5-2-F	GCCAGTGGATGTCCCCATAC
OlRev1-exon5-2-R	TGGTTGGAGCCGGAAGTAAG
OlRev1-exon6-7-F	GGTGCAAAAAGGGTTCAGGA
OlRev1-exon6-7-R	CTCGCTGATGGGATGCTTTT
OlRev1-exon8-9-F	TTTGGTTCATCGTGGTGGTG
OlRev1-exon8-9-R	TGTAACTGTGGAGGGCCAAA
OlRev1-exon10-11-F	GCAAGGTGAGAGGGAGCTGA
OlRev1-exon10-11-R	TAACATGCCCCACCCCTAAA
OlRev1-exon12-13-F	CCTGCTGGCCACAGACATTA
OlRev1-exon12-13-R	CAGTTCATGGCCTAAAAATAGTTGG
OlRev1-exon14-15-F	TTTTCCCCATCCATTTACTTCA
OlRev1-exon14-15-R	TCCAGCACAGAACGATCCAC
OlRev1-exon16-F	CGCCTTCCCAGGTACATCTC
OlRev1-exon16-R	AAGATTTGCTCACGGTTGATGA
OlRev1-exon17-18-F	CTGACTGCACCCAGCTAACA
OlRev1-exon17-18-R	CCATCACAGCAAGACTCACC
OlRev1-exon19-21-F	TGAACTCACGGCTCTCATGG
OlRev1-exon19-21-R	CAAAAACCACATCCGGTGAA
BAC insertion of ActGFP	
Asc042-3'ComplFor	AAGGCGCGCCCTGTAGCGGCGCATT
FseActP5'Rev	CTCTCTGGCCGGCCATGCATTAGTTATTACTAGCGC
Mutation insertion to BAC by Recombineering	
Rev1H489NForRNUP	TTGAACAGACGGGCAGTCTGATCTCCGAGTTCTATTCCCACTCCCGATTGGGCCTGGTGATGATGGCGGGATCG
Rev1H489NRevRNL	AAGTTCATTTATGTATTCAGAAAAGCCGATCCTCCATGTGGAGATCTGGTTCAGAAGAACTCGTCAAGAAGGCG
Rev1H489NFor	TGAACAGACGGGCAGTCTGATCTCCGAGTTCTATTCCCACTCCCGATTGAACCAGATCTCCACATGGAGGATCGGCTTTTCTGAATACATAAATGAACTT
Rev1H489NRev	AAGTTCATTTATGTATTCAGAAAAGCCGATCCTCCATGTGGAGATCTGGTTCAATCGGGAGTGGGAATAGAACTCGGAGATCAGACTGCCCGTCTGTTCA
Rev1L980XForRNUP	TCTTCCACCGGACGGTCGGGGTCCTCGGTCATGCTCCATTAAGGAGATGTGGCCTGGTGATGATGGCGGGATCG
Rev1L980XRevRNL	aaaagtgtacaaacaaattaaactaacTGTTCTTATTGGATCTGATGGTCTCAGAAGAACTCGTCAAGAAGGCG
Rev1L980DelFor	TCTTCCACCGGACGGTCGGGGTCCTCGGTCATGCTCCATTAAGGAGATGTGAaggagcagtccccgtcttcaccggatgtggtttttgcacttctattgt
Rev1L980DelRev	acaatagaagtgcaaaaaccacatccggtgaagacggggactgctcctTCACATCTCCTTAATGGAGCATGACCGAGGACCCCGACCGTCCGGTGGAAGA
pGEX-expression vector Construction	
OlRev1-Bam-ATG	ttaggatccATGAGTCGAGATGGCTGG
OlREV1 -stop-Xho	aatctcgagtcaCGTTATCTTCAGGATGCT
RT−qPCR	
OlRev1_qPCR_5F	GCTTTCACAACGGCTCAGTATC
OlRev1_qPCR_5R	TCTTCCTCATCCAGCCATCTC
OlATM_qPCR_5F	TGCAGAGGGCTGGAACATGATA
OlATM_qPCR_5R	CCTGGTCCTGAAGACATTGTCC
OlMGMT_qPCR_5F	GTCAGCGGATGTGAGAATGG

OlMGMT_qPCR_5R	CGTTTCTGCTGGGCTATCG
OlMGMT_qPCR_3F	AACGCCTCGCTGAAATGG
OlMGMT_qPCR_3R	CCTTGCCGCTCATGTATGG
OlbActin_qPCR_3F	GAGCGTGGCTACTCCTTCAC
OlbActin_qPCR_3R	AGCACAGTGTTGGCGTACAG
