# Supplementary material for: Involvement of Rev1 in alkylating agent‐induced loss of heterozygosity in Oryzias latipes
Source: Genes Cells. 2020 Feb 5;25(2):124–38. doi: 10.1111/gtc.12746 (PMC7079036; doi:10.1111/gtc.12746)
Supplement: Supplementary file 3 [file GTC-25-124-s003.pdf]

	Gender	Number of Individual: Test Specimen	Survived Fish	Tumor-bearing
Un-treated				
Wild	Female	8	7	0
	Male	11	11	1
R530X	Female	12	12	0
	Male	12	9	0
L980X	Female	9	8	0
	Male	16	15	0
H489N	Female	9	9	0
	Male	20	20	0
WildTg	Female	11	11	0
	Male	5	3	0
L980XTg	Female	12	9	0
	Male	11	7	0
H489NTg	Female	15	13	0
	Male	15	9	0
DENA-treated				
Wild	Female	13	12	1
	Male	16	15	4
R530X	Female	18	3	0
	Male	18	6	0
L980X	Female	16	4	0
	Male	20	1	0
H489N	Female	12	10	4
	Male	18	17	12
WildTg	Female	12	11	0
	Male	5	4	0
L980XTg	Female	15	4	0
	Male	15	3	0
H489NTg	Female	15	7	0
	Male	15	12	4

Table S2. Summary of the numbers of male and female fish used, survived and tumor-bearing. Tumor incidence was more frequent in male than female, although the gender difference is not statistically significant. Our result is consistent with the report by Nakazawa et al.(1985), but differs from some reports. Brown-Peterson et al. (1999) found neoplastic liver development did not differ between male and female medaka. In contrast, Teh and Hinton(1998) found female medaka had a significantly greater incidence of hepatic neoplasia. The difference in results could be related to the differences in DENA-exposure protocols as well as the age of the medaka at exposure as suggested by Brown-Peterson et al. (1999). We exposed 4 months-old-medaka to 60 ppm DENA for 2 weeks. Nakazawa et al. (1985) exposed 1 year-old-medaka to 50 ppm DENA for 6 weeks. Teh and Hinton (1998) exposed 21 day-old-medaka to 250 ppm DENA for 48 hr. Brown-Peterson et al. (1999) exposed 14 days-old-medaka to 10-100 ppm DENA for 48 hr. Perhaps there is a differential susceptibility to neoplasia by gender in medaka that is related to a combination of age at exposure as well as to the dosage and duration of exposure to DENA.
